# Sorting of cells of the same size, shape, and cell cycle stage for a single cell level assay without staining

**DOI:** 10.1186/1471-2121-7-25

**Published:** 2006-06-22

**Authors:** Kiyoshi Ohnuma, Tetsuya Yomo, Makoto Asashima, Kunihiko Kaneko

**Affiliations:** 1 Graduate School of Arts and Science, University of Tokyo, 3-8-1 Komaba, Meguro, Tokyo 153-8902, Japan; 2 Department of Biotechnology, Graduate School of Engineering, Osaka University, 2-1 Yamadaoka, Suita, Osaka 565-0871, Japan; 3 ERATO project, Japan Science and Technology Corporation (JST), Tokyo, Japan; 4 ICORP project, Japan Science and Technology Corporation (JST), Tokyo, Japan

## Abstract

**Background:**

Single-cell level studies are being used increasingly to measure cell properties not directly observable in a cell population. High-performance data acquisition systems for such studies have, by necessity, developed in synchrony. However, improvements in sample purification techniques are also required to reveal new phenomena. Here we assessed a cell sorter as a sample-pretreatment tool for a single-cell level assay. A cell sorter is routinely used for selecting one type of cells from a heterogeneous mixture of cells using specific fluorescence labels. In this case, we wanted to select cells of exactly the same size, shape, and cell-cycle stage from a population, without using a specific fluorescence label.

**Results:**

We used four light scatter parameters: the peak height and area of the forward scatter (*FSheight *and *FSarea*) and side scatter (*SSheight *and *SSarea*). The rat pheochromocytoma PC12 cell line, a neuronal cell line, was used for all experiments. The living cells concentrated in the high *FSarea *and middle *SSheight/SSarea *fractions. Single cells without cell clumps were concentrated in the low SS and middle FS fractions, and in the higher *FSheight/FSarea *and *SSheight/SSarea *fractions. The cell populations from these viable, single-cell-rich fractions were divided into twelve subfractions based on their *FSarea-SSarea *profiles, for more detailed analysis. We found that *SSarea *was proportional to the cell volume and the *FSarea *correlated with cell roundness and elongation, as well as with the level of DNA in the cell. To test the method and to characterize the basic properties of the isolated single cells, sorted cells were cultured in separate wells. The cells in all subfractions survived, proliferated and differentiated normally, suggesting that there was no serious damage. The smallest, roundest, and smoothest cells had the highest viability. There was no correlation between proliferation and differentiation. NGF increases cell viability but decreases the proliferative ability of the PC12 cells.

**Conclusion:**

We demonstrated a pretreatment method to collect well-characterized, viable, single cells without using fluorescent labels and without significant damage to the cells. This method is quantitative, rapid, single-step, and yields cells of high purity, making it applicable for a variety of single-cell level analyses.

## Background

Recent technical developments have enabled many phenomena to be detected at the single-cell level. For example, improved amplification of RT-PCR now enables single-cell analysis, while the micro-fabrication technique allows better control of cell positioning, morphological analysis, and examination of intercellular contacts between cells [1-3]. Conventional single-cell level measurement techniques such as patch clamping and time-lapse microscopy have also been improved by increasing the number of simultaneous measuring points, thus enabling high-throughput measurement without specialist technical knowledge [4].

Despite these advances, the simple application of these techniques to a conventional sample is not sufficient. Although heterogeneity within a cell population may have a negligibly small effect on the analysis in population measurements because of population averaging, it becomes prominent in single-cell level analysis. Thus, improvements in sample purification as well as measurement techniques are required for revealing new phenomena. Because a clonal cell line consists of genetically identical cells, it is suitable for single-cell assays. However, properties within a clonal line are not truly homogeneous because of differences in cell cycle, phenotypic variance, and cell damage caused by manipulations. Selection of cells based on empirical knowledge is necessary for meaningful data acquisition at the single-cell level, despite the bias that might be introduced by such a selection process. Thus, a quantitative and rapid sample-pretreatment method of selecting well-characterized and homogeneous cells from a cell line is important for high-quality data acquisition in single-cell assays.

A cell sorter is suitable for the quantitative and high-speed manipulation of individual cells and can be used to separate a specific type of cell based on a composite of flow cytometry (FCM) parameters. A cell sorter is routinely used to measure and sort particular type of cells from dispersed tissue or other mixtures of cell types. Cells can be measured and sorted using both fluorescent labels and light scattering. Measuring the fluorescence of a specific label is useful because labels can be used to amplify cell features. However, the labelling will be different for each application and for each sample. Moreover, labeling is not uniform and may even damage the cells. On the other hand, light scattering reflects general cell properties such as size and shape [5,6], but is free from the above disadvantages.

In the present study, we applied a cell sorter to select cells of the same size, shape, and cell-cycle stage, based on light scattering parameters. We used both peak height and area (integrated value) of light scatter parameters (Fig. 2), of which only one was generally used to sort cells, and did not use a specific label. We used the rat adrenal pheochromocytoma PC12 cell line, which behaves like neural progenitor cells; the cells proliferate in serum-supplemented media and differentiate into sympathetic neuron-like cells with long neurites upon the addition of nerve growth factor (NGF) [7]. The PC12 cells are easy to handle and culture, can survive when single cells are isolated in a separate well, and neuronally differentiated cells are readily distinguished under the microscope. At first, fractions rich in living cells and single cells were identified. Then, the single live-cell-rich region was divided into twelve subfractions, and the size and shape of the cells and cell phase in each fraction were correlated using light scattering parameters. Finally, to test the methods as well as to characterize the basic properties of sorted cells at a single-cell level, single cells were sorted from each subfraction into individual wells of a 96-well plate to determine the viability, proliferation, and differentiation potential with or without NGF.

**Figure 1:**
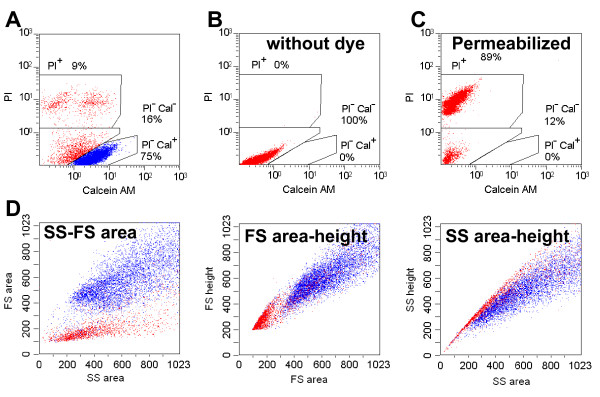
**Living and dead cell fractions**. **A **and **D: **two-dimensional profiles of the PC12 cells stained with calcein AM and PI. Calcein-PI (**A**) and *SSarea-FSarea *(**D**, left), *FSarea-FSheight *(**D**, middle) and *SSarea-SSheight *(**D**, right). Cells without dye (**B**) and cells permeabilized by 0.1 % saponin with dye added (**C**) were used to define the dead cells. Calcein-positive and PI-negative cells (living cells) are represented by blue dots and calcein-negative or PI-positive cells (dead cells) are represented by red dots.

**Figure 2:**
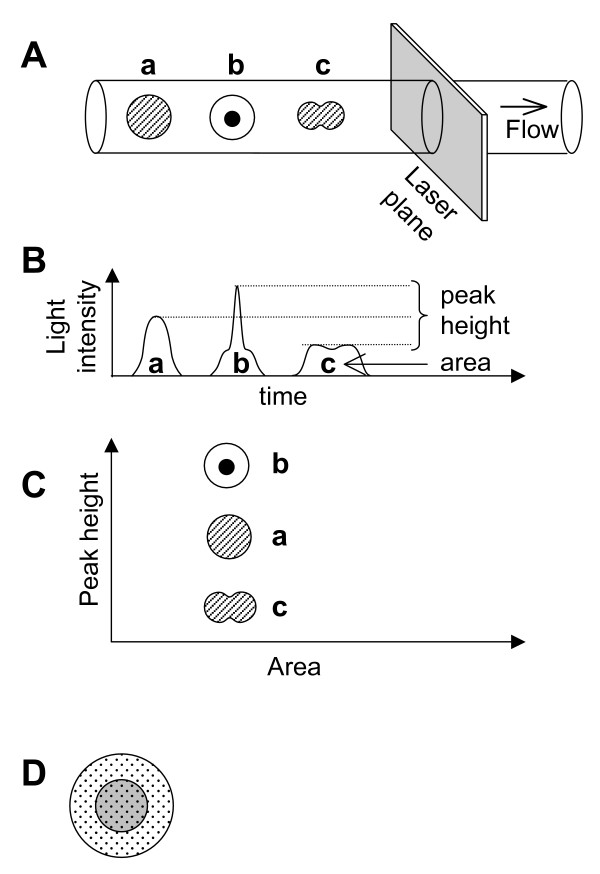
**Schematic representation of the particle structures, with peak height and area**. **A: **Single cell with uniform content (a), single cell without uniform content (b), and doublet cells with uniform content (c). **B: **Time course of signals. **C: **Position of the cells in the height and area profiles. **D: **A sphere consists of uniformly dispersed small reflectors and has a central part with a slightly different refractive index (see Discussion).

## Results

### Living cell fractions

To determine the fractions containing living cells by light scattering, PC12 cells were stained with the fluorescent markers calcein AM and propidium iodide (PI). Calcein AM is a fluorogenic esterase substrate that is hydrolyzed to a green fluorescent product in living cells; thus, green fluorescence was an indicator of cells with esterase activity as well as an intact membrane to retain the esterase products. PI is a red fluorescent nucleic acid stain that is only able to pass through the compromised membranes, thus it marks dead cells. In Fig. 1, each blue dot identifies the calcein-positive and PI-negative cells as living cells, whereas each red dot represents calcein-negative or PI-positive cells, which were assumed to be dead cells.

The fact that fractions containing red and blue dots were clearly separated in the scatter profiles in Fig. 1 suggests that using four light scatter parameter enables better gating out of the dead cell. As expected, the red dots were concentrated in the low *FSarea *fraction and the low to medium *SSarea *fractions, which may contain cell fragments. We found that red dots were also concentrated in the upper fraction of *FSarea*-*FSheight *profile and especially in the upper fraction of the *SSarea*-*SSheight *profile. Because dead cells may contain structures with increased light scattering properties, such as vacuoles or aggregated proteins, the height-to-area signal ratio in this fraction was high (see Fig. 2).

### Single-cell fractions

It is difficult to completely disperse cells mechanically to obtain truly single cells; usually the dispersed suspension contains many cell aggregates. Extensive enzyme digestion and trituration may aid in breaking up cell clumps, but will tend to cause cell damage. In addition, small parts of the cells may re-aggregate. When the PC12 cells were dispersed using trituration by pipette in calcium- and magnesium-free phosphate-buffered saline (PBS^-^), more than half of the cells remained as aggregates (see also [8]). Filtration and density-gradient centrifugation are easy way to remove big aggregates, and cell aggregates can be discriminated using a cell sorter to gate out particles that have a large peak and/or prolonged (broad) signal. However, these methods cannot be used to distinguish between cell doublets and large single cells. Here, we hypothesized that a cell doublet may exhibit a lower height-to-area signal ratio than a single cell of the same size as shown in Fig. 2. Although the longer axis of the doublet will not always be positioned parallel to the flow, the accuracy of doublet detection can be increased by using both FS and SS, which are at right angles to each other.

**Figure 3:**
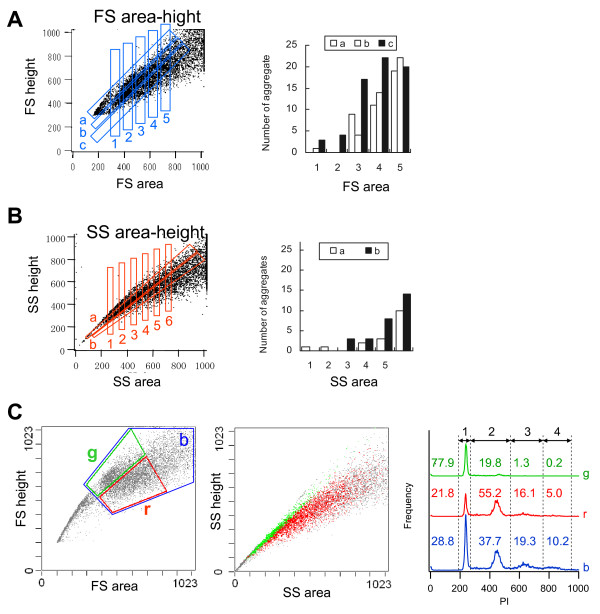
**Single and aggregate cell fractions**. **A, B: **Number of aggregates in live cells, which were sorted according to their FS profile (**A**) and SS profile (**B**). **A: **Fifty gates in the *FSarea-FSheight *profile (left). Particles in each cross-section of gates a-c and gates 1–5 in the *FSarea-FSheight *profile were sorted. Then, the nuclei per particle were counted, and the number of cell aggregates per twenty-seven particles is shown (right). **B: **Eleven gates in the *SSarea-SSheight *profile (left), and the number of aggregates per twenty-seven particles (right). **C: ***FSarea-FSheight *(left), *SSarea-SSheight *(middle), and PI (right) profiles of the ethanol-fixed cells. The green and red dots shown in the *SSarea-SSheight *profile are the cells within the gate *g *and *r *shown in the *FSarea-FSheight *profile, respectively. Traces *g*, *r *and *b *in the PI profile correspond to gates *g*, *r *and *b *in the *FSarea-FSheight *profile, respectively. The numbers represent the percentage of cells in regions 1–4.

We tested this working hypothesis by sorting cells based on the *FSarea*-*FSheight *profile or the *SSarea*-*SSheight *profile. The nuclei of the sorted cells were then stained with SYTO24, a cell-permeable green fluorescent nucleic acid stain, and the nuclei in each particle were counted. Fig. 3A shows the *FSarea*-*FSheight *results. The single cell ratio decreased as *FSarea *increased. However, even among low *FSarea *fractions, the lower *FSheight*-to-*FSarea *ratio fractions contained larger numbers of doublets. The same findings were also seen in the *SSarea*-*SSheight *profiles (Fig. 3B). Although late M phase cells were counted as doublets using this method, this limitation is unlikely to have a large impact on the results. We concluded that our working hypothesis was correct.

We further tested the hypothesis using fixed cells. Ethanol-fixed cells were stained with PI and analyzed for relative DNA content using FCM. Although the scatter profiles of these cells (Fig. 3C) were not identical to that of the living cells (Fig. 1D), the scatter profiles had roughly the same shape. In trace *b *of the PI profile (Fig. 3C), which corresponds to fraction *b *of the *FSarea*-*FSheight *profile, the first peak at 230 and the second peak at 460 corresponded to the G1 and M cell cycle phases, respectively. However, there were additional small peaks around 650 and 850, which positioned at about three and four times the fluorescence of the first peak, respectively. These peaks may correspond to cell aggregates. In fact, the lower *FSheight*-to-*FSarea *ratio fraction (*r*) contained 17% cells around the two extra peaks, but the higher *FSheight*-to-*FSarea *ratio fraction (*g*) contained less than 2% of cells around the third and fourth peaks. In addition, the cells in the higher *FSheight*-to-*FSarea *ratio fraction were in the higher *SSheight*-to-*SSarea *ratio fraction (Fig. 3C). We concluded again that our working hypothesis about the single cell fraction and light scattering was correct. These results suggest that single cells can be separated without using a specific dye, as they were abundant in low area-signal fractions and higher height-to-area signal ratio fractions. These fractions were both useful for gating out doublets.

### Twelve single live-cell subfractions in the *FSarea-SSarea *profile

**Figure 4:**
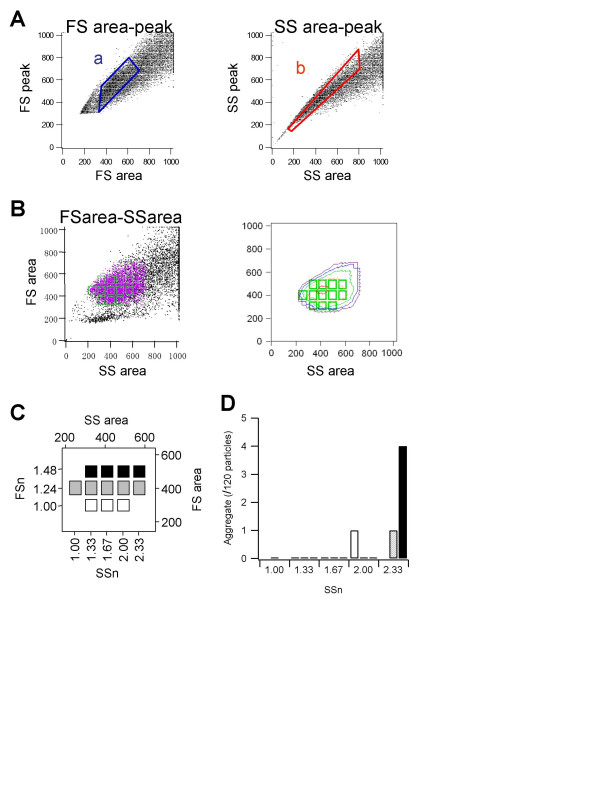
**Living- and single-cell gates and the twelve subfractions**. **A: **Living and single cell-rich gates in *FSarea-FSheight *(a, blue) and *SSarea-SSheight *(b, red) are shown. **B: ***SSarea-FSarea *profile. Left: dot plot of all cells (black) and cells in the *a *and *b *gates (purple dots). Right: contour plot of the cells in the *a *and *b *gates. The twelve sorting gates are represented by the green rectangles. **C: **Schematic representation of the twelve gates. *SSarea *and *FSarea *values were normalized to the smallest value and represented as *SSn *and *FSn*. **D: **The number of aggregate cells in the twelve subfractions (n = 120). The SYTO24-stained nuclei in the sorted cells were counted. Shading corresponds to that in C.

Based on the results thus far, we determined the fractions containing live single cells in the *FSarea*-*FSheight *and *SSarea*-*SSheight *profiles (Fig. 4A). Because the shape of gates *a *and *b *were almost linear, the four parameters could be represented by two parameters. We chose *FSarea *and *SSarea*. Violet dots in Fig. 4B indicate the cells in gates *a *and *b*. Twelve gates were then set within the gated *FSarea-SSarea *profile (Fig. 4B). These twelve gates were represented by their respective, normalized *FSarea *and *SSarea *coordinates (*FSn*, *SSn*), as shown in Fig. 4C. Live cell ratios based on Fig. 1 for all twelve fractions were more than 80%. The low *FSn *and high *SSn *fractions contained slightly fewer living cells than the other subfractions. However, all subfractions were thought to mainly contain living cells. To verify that each subfraction consisted of single cells, the subfractions were counted using syto24 (Fig. 4D). One hundred and twenty cells in each fraction were sorted, and the number of doublets counted. Although a few (less than 4%) doublets or single cells in M phase appeared in the large *SSn *fractions, their number was small enough to be negligible. These results suggest that the twelve subfractions consisted of single cells.

### Size and shape of cells in the twelve subfractions

**Figure 5:**
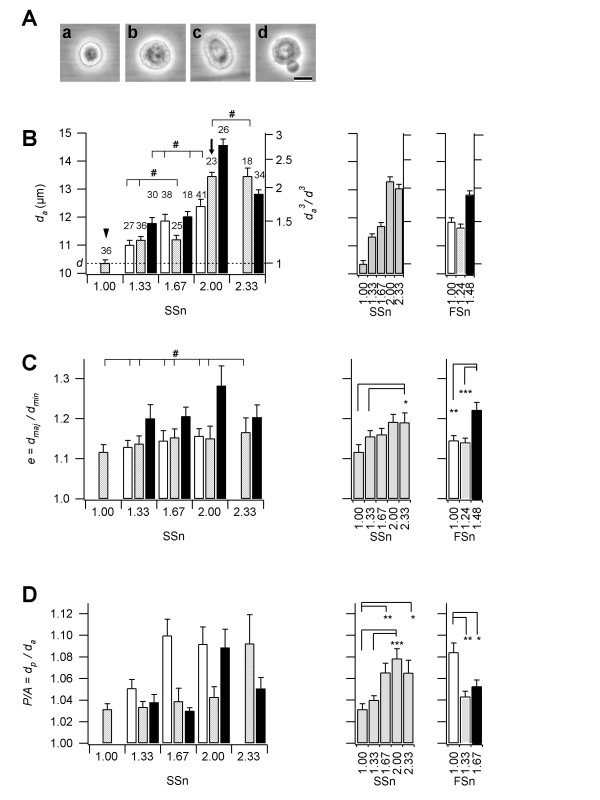
**Cell size and shape of the sorted cells in the twelve subfractions**. **A: **Phase-contrast micrographs of the sorted cells: spherical and small (**a**); spherical and large (**b**); oval (**c**); irregular surface (**d**). **B-D: **The diameter (*d*_*a*_, **B**), elongation (*e*, **C**), and surface roughness (*P/A*, **D**) of the cells are shown. These values were evaluated from the phase-contrast images of cells (see Methods). Left, average for each subfraction; middle, average for *SSn*; right, average for *FSn*. Shading corresponds to that in Fig. 4C. The number of measured cells is shown on each column in **B**. The right ordinate of **B **is the relative cell volume (*d*_*a*_^3^/*d*^3^), where *d *is the average of *d*_*a *_at (*SSn*, *FSn*) = (1.00, 1.24) (arrow head). The relative cell volume at (*SSn*, *FSn*) = (2.00, 1.24) (arrow) was about two. Statistically significant for **C **and **D **at *P < 0.05; **P < 0.005; ***P < 0.0005. Statistically not significant for **B **and **C **at #*P *> 0.05.

We next attempted to correlate light scattering parameters with cell microscopy. The subfraction cells were sorted and floating cells were imaged by phase contrast microscopy to reveal many cell types (Fig. 5A). We focused on three characteristics: diameter (*d*_*a*_), elongation (*e*), and surface roughness (*P/A*) (see Methods).

As shown in Fig. 5B, the cell diameter increased with the increasing *SSn *and *FSn *values. We fit the *d*_*a *_as a function of *SSn *and *FSn*. The fitting function is as follows,

da∝(SSn)1a(FSn)1b
 MathType@MTEF@5@5@+=feaafiart1ev1aaatCvAUfKttLearuWrP9MDH5MBPbIqV92AaeXatLxBI9gBaebbnrfifHhDYfgasaacH8akY=wiFfYdH8Gipec8Eeeu0xXdbba9frFj0=OqFfea0dXdd9vqai=hGuQ8kuc9pgc9s8qqaq=dirpe0xb9q8qiLsFr0=vr0=vr0dc8meaabaqaciaacaGaaeqabaqabeGadaaakeaacqWGKbazdaWgaaWcbaGaemyyaegabeaakiabg2Hi1kabcIcaOiabdofatjabdofatjabd6gaUjabcMcaPmaaCaaaleqabaWaaSaaaeaacqaIXaqmaeaacqWGHbqyaaaaaOGaeiikaGIaemOrayKaem4uamLaemOBa4MaeiykaKYaaWbaaSqabeaadaWcaaqaaiabigdaXaqaaiabdkgaIbaaaaaaaa@40CA@

where *a *and *b *were the fitting parameters. The *a *value was 3.2, suggesting that *SSn *has a linear relationship with cell volume. Actually, the cell volume at (*SSn*, *FSn*) = (2.00, 1.24) was about twice as large as that at *(SSn*, *FSn*) = (1.00, 1.24) as shown in Fig. 5B. The *b *value was 6.8, suggesting that *FSn *has a weaker positive correlation with cell diameter. Elongation (*e*) also increased with the increasing *SSn *and *FSn *(Fig. 5C), but depended more on *FSn *than on *SSn*. The *e *at the largest *SSn *is larger than at other values. At middle and low *FSn*, although the *d*_*a *_were quite different, all the *e *were the same (*P *> 0.1) suggest that the larger cells are not necessarily the elongated cells. There may be two different mechanisms between volume and elongation.

The roughness of the cell surface, *P/A*, exhibited different dependencies from the cell diameter and elongation, increasing with *SSn *but decreasing with *FSn*. The cell, which had a high *P/A *value, had bubbles around it (Fig. 5Ad) and appeared non-viable. The Pearson's correlation coefficient for the *P/A *value compared to the ratio of calcein-positive and PI-negative cells (living cells) in the twelve subfractions was significantly smaller than zero (*p *< 0.05) also supports the view.

### Cell cycle in the twelve subfractions

**Figure 6:**
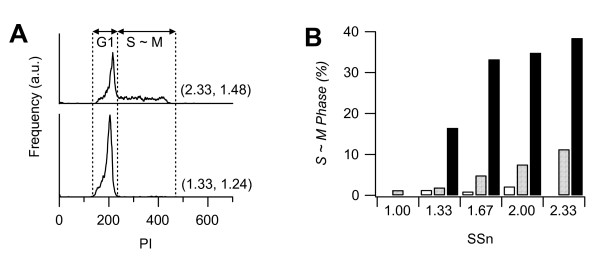
**Cell cycle of the sorted cells in the twelve subfractions**. Live cells were sorted according to the twelve subfractions, then fixed and stained with PI. **A: **Two examples of PI profile. Upper trace: (*SSn*, *FSn*) = (2.33, 1. 48), lower trace: (1.33, 1.24). The left peak corresponds to the G1 phase and the right region corresponds to S, G2 and M phase. **B: **Percentage of S, G2 and M phase cells. Shading corresponds to that in Fig. 4C.

Next, we examined the association between light scattering and cell cycle. Because light scattering profiles have been closely correlated to nuclear morphology [9,10], this method may also be useful in distinguishing cells in a specific stage of the cell cycle. The PC12 cells in the twelve subfractions were sorted then fixed and stained with PI. The relative DNA contents in the subfractions were measured again by FCM. Fig. 6A shows examples of PI profiles in two subfractions. The lower *SSn *and *FSn *subfractions (1.33, 1.24) contained only G1 phase cells but the higher *SSn *and *FSn *subfraction (2.33, 1.48) contained many S ~ M phase cells. Fig. 6B shows the percentage of S ~ M phase cells in all subfractions. Although the percentage increased with both increasing *SSn *and *FSn*, the percentage at the largest *SSn *is apparently larger than at other values. This distribution was the same as observed for elongation (Fig. 5C). In fact, the Pearson's correlation coefficient between the ratio of cells in S ~ M phase and elongation was 0.9 (significantly positive at *P *< 0.0025), indicating that elongated cells are in S ~ M phase and abundant in the high *FSn *fractions. It is surprising that the Pearson's correlation coefficient between the ratio of cells in S ~ M phase and cell size was not significantly positive (P > 0.05). Although the cell volume at (*SSn*, *FSn*) = (2.00, 1.24) was twice as large as that at *(SSn*, *FSn*) = (1.00, 1.24), more than 90% of cells in both subfractions were in the G1 phase. One might expect a specific cell volume at which the cells started to synthesize DNA, however this was not the case. The results suggested that PC12 cells in G1 phase are spherical even if the cell volume has increased, and then the cells move into S phase and start to elongate.

### Single cell assay in the twelve subfractions

We performed a fundamental single cell assay to verify our sorting method developed for doing single-cell assays, and to determine the fundamental properties of single cells. The single sorted cells of all twelve subfractions were cultured in separate wells of a 96-well plate and tested for viability, proliferation, and differentiation potential.

**Figure 7:**
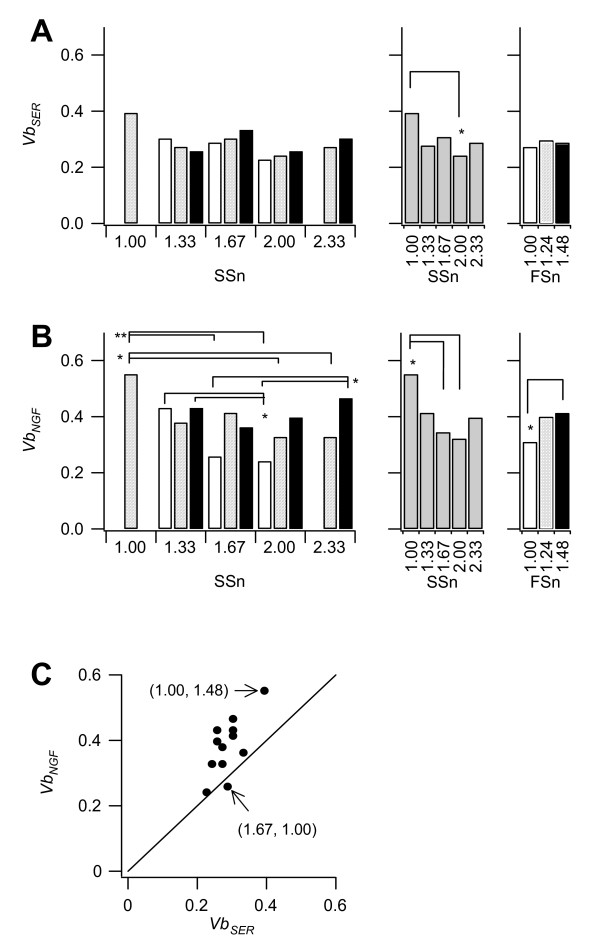
**Viabilities of single-sorted cells plated in separate wells**. Cell viability in each of the twelve sub-fractions with (**B**) and without (**A**) NGF. Left, average for each subfraction; middle, average for *SSn*; right, average for *FSn*. Shading corresponds to that in Fig. 4C. Statistically significant at *P < 0.05; **P < 0.005. **C: **SER versus NGF for each subfraction. The slope of the line is 1.

The viability (*Vb*) of cells from all subfractions was higher than 20% and up to 60% in the presence of NGF (Fig. 7). Because it is well known that viability is low with low concentration culture, the observed viabilities were higher than expected. The differentiation ratio of survived cells was less than 3% without NGF but more than 70% with NGF in all subfractions. The survived cells divided more than five times without NGF but less than 3 times with NGF within 13 days. The sorted single PC12 cells behaved normally; they proliferated readily in medium without NGF but proliferation slowed and differentiation into neuronal cells was frequent in medium containing NGF [7]. These results suggest that the cells responded normally to NGF and therefore sorting and single-cell culturing do not introduce serious damage to the cells.

One might expect a negative correlation between proliferation and differentiation potential in these cells; however this was not the case. The differentiation ratio and proliferation rate were similar in all subfractions. It is notable that viability at subfraction (1.00, 1.24) was significantly higher than for the other subfractions (Fig. 7C). Because this subfraction contained the smallest, roundest, and smoothest cells (Fig. 5), this was an intuitive result. It is also notable that the cells with NGF showed high viability but a low proliferation rate. The *Vb*_NGF _values were significantly larger than the *Vb*_SER _values (*P *< 0.05), and the average *Vb*_NGF _to *Vb*_SER _ratio was 1.3 (Fig. 7C). Although the addition of NGF to serum-free medium, in which the cells can not survive, dramatically improves cell viability [11], the effect of adding NGF to serum-supplemented medium, in which the cells can survive and proliferate, has not been measured. Generally, cell viability assays are performed using mass cultures, and viability factors cannot be distinguished from proliferation factors. On the contrary, our single cell assays can evaluate viability and proliferation independently. We demonstrated directly that NGF increases cell viability but decreases the proliferative ability of the PC12 cells when cultured in the presence of serum.

## Discussion

This study demonstrated a non-staining sample-pretreatment method to select well-characterized homogeneous cells from a cell line for single-cell level analysis. Although a cell line is genetically identical, it is morphologically heterogeneous due to aggregation, cell cycle, and phenotype variance. We used both peak height and area of light scattering parameters to sort PC12 cells into live single-cell fractions, without the use of a specific label. Although it is usually difficult to discriminate between single large cells and small cell aggregates, we successfully separated these classes using the height-to-area signal ratio of both FS and SS. The distinction methods were versatile enough to apply to both living and ethanol-fixed cells. Moreover, this discrimination method is applicable not only to pretreatment steps but also in the data acquisition stage.

We showed that the *FSn *is closely related to both the degree of cell elongation and the amount of DNA per cell, and that the *SSn *was proportional to the cell volume. This is in contrast to the commonly accepted association between light scattering and cell morphology that FS represents the size and SS represents internal structure (cf. owner manual of ALTRA). Light scattering profiles are closely related to morphological features including size, shape, surface detail, and intracellular structure of the particle [6,9,10,12]. The dependency, however, varies according to the structure of the particles and the acquisition method applied [9,13]. The commonly accepted association was based mainly on the analysis of different-size beads or different kinds of hemocyte using narrow angle analysis [9,10]. However, current machines use wide angles; for example, the FS and SS of ALTRA are the summation of light scattering data from -23° to 23° and from 42° to 138° from the laser axis, respectively. Moreover, we tested an adherent cell line, in which the component cells are almost of the same structure and size. Thus, it is not surprising that our results differed from the accepted association. One possible explanation of our results is that the refractive index of the cell nuclei differs slightly from that of the cytosol and that the cell contains many small particles that reflect light and are distributed homogeneously (Fig. 2D). Diffraction and refraction are the main components of forward scatter and thus are affected by the size of the nucleus, which is roughly in proportion to the DNA content of the cell. On the other hand, reflection from small particles is the main component of side scatter. Because *SSn *or *SSarea *is the time integration of side scatter, it is proportional to the number of particles in the cell and as a consequence, is proportional to cell volume.

Light scattering can be used to gain quantitative criteria of cell appearance, which is a good indicator of cell properties. In experiments where data are acquired using a microscope, such as patch clamping, cells must be selected based on appearance to acquire meaningful data. Based on microscopic observation, cell aggregates, dead cells, and less vital cells can be excluded and cells from which data can be acquired, are selected. This empirical selection, however, may introduce an unknown bias to the experiments. Our single-cell assay showed that the roundest and smoothest cells have high viability. This intuitive result suggests that cell selection using light scattering data is consistent with empirical selection based on cell appearance, but without introducing unknown bias.

In the single-cell assay we used the auto-clone option. Because the conventional limiting dilution method is stochastic, there is no guarantee that individual cells are in separate wells. However, auto-clone enabled us to plate a precise number of cells directly into a separate well. Thus, this option would also be useful for single-cell assays of cell extracts, such as single-cell RT-PCR. This option also enabled us to assess cell viability (survival ratio) and cell proliferation independently, which is difficult to achieve in conventional mass culture. Finally, the single-living-cell assay results confirmed that the cell sorting method used in this study caused no significant damage to the cells, as the sorted single cells in the individual wells survived, proliferated and differentiated normally.

## Conclusion

We first demonstrated here that the live single-cell can be distinguished from cell doublets or aggregates from the height-to-area signal ratio in the light scatter parameter. We also demonstrated that the cell volume is estimated by *SSarea*, while the elongation and the level of DNA in the cells is estimated by using *FSarea*, which suggests that elongation of cell occurs at the late stage of cell cycle. Then we performed a single cell assay using auto-clone option. The single isolated cells survived, proliferated and differentiated normally, suggesting that no serious damage was caused by the method. We also show the relationship among basic properties of cells such as morphology, cell cycle, viability, proliferation and differentiation. Viability of a cell is estimated from the scattering data separately from the proliferation.

These results suggest that using a cell sorter as a pretreatment method for single-cell assay has several significant advantages. First, the light scatter profiles provide quantitative indicators of microscopic cell appearance, thereby reflecting empirical selection without introducing unknown bias. Moreover, fluorescent dyes such as GFP can be included into the technique if required. Second, because the cells can be sorted based on DNA content and fine differences in cell size, cell volume dependent characters, such as whole-cell expression levels and currents, can be measured with low variance. Third, the auto-clone option enables an exact number of selected cells to be plated into separate wells. Finally, all of these treatments can be done at once and is rapid and less invasive to the cells. Thus, cell sorting as used in this study provides a valuable pre-treatment step for single-cell level assays. This method can be applied not only to various types of single-cell assay but also to single-cell level analysis to investigate complex systems, such as the dynamics of multicellular interactions in which a small difference in the initial state can cause a large difference in the final state.

## Methods

### Cell culture

PC12 cells were obtained from the Riken Cell Bank (Cat #: RCB0009; Ibaragi, Japan). PC12 cells were cultured according to the method described by Green et al. [8]. Briefly, PC12 cells were grown on collagen I-coated plastic dishes (Biocoat; Becton Dickinson, NJ, USA) at 37°C in a humidified incubator in an atmosphere of 5% CO_2_. The normal culture medium consisted of 85% Dulbecco's modified Eagles' medium (DMEM; Invitrogen Corporation, Carlsbad, CA, USA), 10% heat-inactivated horse serum (Invitrogen), 5% heat-inactivated newborn calf serum (Invitrogen), 50 units/ml penicillin and 50 μg/ml streptomycin (Invitrogen), and 4 mM L-glutamine (Invitrogen).

### Flow cytometry and cell sorting

The culture medium was first replaced with ice-cold calcium- and magnesium-free phosphate-buffered saline (PBS^-^) containing 1 mg/ml bovine serum albumin (BSA), then the cells were detached from the dish by forceful aspiration of the solution through a 1-ml plastic pipette. The cell suspension was then filtered through a 40-μm nylon mesh (Becton Dickinson) to remove large cell aggregates. Preliminary experiments showed that the FCM parameters depended on temperature, therefore all solutions and samples were kept on ice during subsequent procedures. In experiments that included cell staining, fluorescent dyes were added to the suspension at this point. Preliminary experiments showed that the FCM profile drifted about 20 minutes after harvesting and became relatively stable thereafter. Thus, the suspension was kept ice cold for 30 minutes after harvesting, and then FCM acquisition and cell sorting were performed within 40 minutes using an EPICS ALTRA (Beckman Coulter, Inc., Miami, FL, USA). All measurements were made using an argon laser at 488 nm. The beam size at the focal plane was 6 μm in height and 100 μm in width. The cells were sorted according to four parameters, which comprised the peak height and area of forward scatter (*FSheight *and *FSarea*) and side scatter (*SSheight *and *SSarea*). The peak shape (peak width or time of flight), which is the transit time of a particle in the laser beam plane, is also a basic parameter. However we did not use the peak shapes because it was sensitive to the operation and was relatively unstable. PMT 2 was used to measure the SS because it had both area- and peak-value output.

The cell sorting settings were as follows: flow chip diameter, 100 μm; sheath solution, IsoFlow (Beckman Coulter); sheath pressure, 8.8 – 9.2 PSI; crystal frequency, 19.0 – 21.5 kHz; crystal drive, 15–30%; delay, 25 – 32 drops; sort mode, 3; coincident abort, on; and pulse pile up (PPU) sensitivity, 4–10 μs.

### Cell size and shape

To evaluate the size and shape of the cells, phase-contrast images were captured using a digital camera (Coolpix 995; Nikon, Tokyo, Japan) placed on an inverted microscope (TS100; Nikon). After importing the images to a computer, an outline of each cell image was traced by hand. The perimeter (*P*), area (*A*), and the major and minor axes of the best-fitted ellipse (*d*_*maj *_and *d*_*min*_) for the outlined cells were then measured using image analysis software (Scion Image; Scion Corporation, Frederick, ML, USA).

To estimate the cell diameter, *d*_*maj*_, *d*_*min *_and the equivalent circular diameter (dp≡P2π
 MathType@MTEF@5@5@+=feaafiart1ev1aaatCvAUfKttLearuWrP9MDH5MBPbIqV92AaeXatLxBI9gBaebbnrfifHhDYfgasaacH8akY=wiFfYdH8Gipec8Eeeu0xXdbba9frFj0=OqFfea0dXdd9vqai=hGuQ8kuc9pgc9s8qqaq=dirpe0xb9q8qiLsFr0=vr0=vr0dc8meaabaqaciaacaGaaeqabaqabeGadaaakeaacqWGKbazdaWgaaWcbaGaemiCaahabeaakiabggMi6oaalaaabaGaemiuaafabaGaeGOmaidcciGae8hWdahaaaaa@3554@) and equivalent area diameter (da≡Aπ
 MathType@MTEF@5@5@+=feaafiart1ev1aaatCvAUfKttLearuWrP9MDH5MBPbIqV92AaeXatLxBI9gBaebbnrfifHhDYfgasaacH8akY=wiFfYdH8Gipec8Eeeu0xXdbba9frFj0=OqFfea0dXdd9vqai=hGuQ8kuc9pgc9s8qqaq=dirpe0xb9q8qiLsFr0=vr0=vr0dc8meaabaqaciaacaGaaeqabaqabeGadaaakeaacqWGKbazdaWgaaWcbaGaemyyaegabeaakiabggMi6oaakaaabaWaaSaaaeaacqWGbbqqaeaaiiGacqWFapaCaaaaleqaaaaa@3441@) were used. However, because these four values had a strong correlation (Pearson's correlation coefficients were greater than 0.96), only *d*_*a *_is shown as data. To quantify the cell shape, ellipticity (e≡dmajdmin⁡
 MathType@MTEF@5@5@+=feaafiart1ev1aaatCvAUfKttLearuWrP9MDH5MBPbIqV92AaeXatLxBI9gBaebbnrfifHhDYfgasaacH8akY=wiFfYdH8Gipec8Eeeu0xXdbba9frFj0=OqFfea0dXdd9vqai=hGuQ8kuc9pgc9s8qqaq=dirpe0xb9q8qiLsFr0=vr0=vr0dc8meaabaqaciaacaGaaeqabaqabeGadaaakeaacqWGLbqzcqGHHjIUdaWcaaqaaiabdsgaKnaaBaaaleaacqWGTbqBcqWGHbqycqWGQbGAaeqaaaGcbaGaemizaq2aaSbaaSqaaiGbc2gaTjabcMgaPjabc6gaUbqabaaaaaaa@3B09@) and the perimeter to surface area ratio (P/A≡dpda
 MathType@MTEF@5@5@+=feaafiart1ev1aaatCvAUfKttLearuWrP9MDH5MBPbIqV92AaeXatLxBI9gBaebbnrfifHhDYfgasaacH8akY=wiFfYdH8Gipec8Eeeu0xXdbba9frFj0=OqFfea0dXdd9vqai=hGuQ8kuc9pgc9s8qqaq=dirpe0xb9q8qiLsFr0=vr0=vr0dc8meaabaqaciaacaGaaeqabaqabeGadaaakeaacqWGqbaucqGGVaWlcqWGbbqqcqGHHjIUdaWcaaqaaiabdsgaKnaaBaaaleaacqWGWbaCaeqaaaGcbaGaemizaq2aaSbaaSqaaiabdggaHbqabaaaaaaa@3757@) of the cells were calculated. In the case of a circle, *e *= *P/A *= 1, whereas *e *and *P/A *inequality give *e *≥ 1 and *P/A *≥ 1. In general, *e *is an indicator of the degree of elongation and *P/A *is an indicator of cell surface roughness.

### Cell staining

To determine the fractions containing living cells, 100 nM of Calcein AM (c3100; Molecular Probes, Inc., Eugene, OR, USA) and 100 ng/ml of propidium iodide (PI, A4378; Sigma-Aldrich, St. Louis, MO, USA) were used. Calcein and PI signals were measured using a 525-nm bandpass filter and a 675-nm bandpass filter. Because the Calcein and PI fluorescence overlap was negligible, the fluorescence signals were not compensated.

To counting nuclei, the live cells were stained by 5 μM of Syto 24 (S7559: Molecular Probes, Inc).

To assess the cell cycle stage, total DNA in each lot of PC12 cells was measured using FCM. The cells were suspended in 300 μl of ice-cold PBS^- ^and 700 μl of -20°C ethanol was added to the suspension while vortexing gently. The fixed cells were stored at -80°C. The cells were washed in PBS^- ^and re-suspended in PI staining solution containing 20 μg/ml of PI, 0.1% (v/v) Triton X-100, and 200 μg/ml of DNase-free RNase in PBS^-^. The cells were incubated for 30 minutes at room temperature and were kept ice cold before FCM acquisition. PI signals were measured using a 675-nm bandpass filter.

### Viability, differentiation and proliferation potential of single cells

PC12 cells were divided into fractions according to the twelve-subfraction separation (Fig. 4C). Single cells were plated into the individual wells of a 96-well plate using the auto-clone option of the ALTRA cell sorter. The success rate for plating one cell per well was more than 90%. Each well was precoated with collagen I (Iwaki, Tokyo, Japan) and contained 100 μl of normal culture medium.

Two day after sorting, half of the wells were given an additional 100 μl of culture medium containing 100 ng/ml of NGF without serum, and the other half were given culture medium with serum. Thus, half of the wells contained 50 ng/ml NGF, 5% HS and 2.5% NCS ("NGF" condition), and the other half contained normal culture medium ("SER" condition). Half of the culture medium was changed every 2 to 4 days. The cells were cultured from eleven to thirty days after sorting. Cell viability (*Vb*) or survival ratio was the ratio of the number of wells containing any cells to the number of single-cell plated wells. The differentiation potential for the surviving cells was the average ratio of neurite-bearing cells per total cells in a well. The proliferation potential for the surviving cells was the logarithm of the average cells in a well to the base 2.

## Authors' contributions

KO and TY designed the included experiments. KO performed all of the experimental work and drafted the manuscript. TY, KK, and MA coordinated and supervised the study. All authors read and approved the final manuscript.
